# Variation of SMSI with the Au:Pd Ratio of Bimetallic Nanoparticles on TiO_2_(110)

**DOI:** 10.1007/s11244-017-0854-5

**Published:** 2017-09-27

**Authors:** Richard Gubó, Chi M. Yim, Michael Allan, Chi L. Pang, András Berkó, Geoff Thornton

**Affiliations:** 1 Department of Applied and Environmental Chemistry, University of Szeged, Rerrich B. tér 1., 6720 Szeged, Hungary; 2 ELI-HU Nonprofit Kft, Extreme Light Infrastructure-ALPS, Dugonics tér 13, 6720 Szeged, Hungary; 3 London Centre for Nanotechnology and Chemistry Department, University College London, 20 Gordon Street, London, WC1H 0AJ UK; 4 MTA-SZTE Reaction Kinetics and Surface Chemistry Research Group, Rerrich B. tér 1., 6720 Szeged, Hungary

**Keywords:** Core–shell nanoparticles, Au–Pd alloys, Scanning tunneling microscopy, SMSI, Encapsulation

## Abstract

Au/Pd nanoparticles are important in a number of catalytic processes. Here we investigate the formation of Au–Pd bimetallic nanoparticles on TiO_2_(110) and their susceptibility to encapsulation using scanning tunneling microscopy, as well as Auger spectroscopy and low energy electron diffraction. Sequentially depositing 5 MLE Pd and 1 MLE Au at 298 K followed by annealing to 573 K results in a bimetallic core and Pd shell, with TiO_x_ encapsulation on annealing to ~ 800 K. Further deposition of Au on the pinwheel type TiO_x_ layer results in a template-assisted nucleation of Au nanoclusters, while on the zigzag type TiO_x_ layer no preferential adsorption site of Au was observed. Increasing the Au:Pd ratio to 3 MLE Pd and 2 MLE Au results in nanoparticles that are enriched in Au at their surface, which exhibit a strong resistance towards encapsulation. Hence the degree of encapsulation of the nanoparticles during sintering can be controlled by tuning the Au:Pd ratio.

## Introduction

Atomically dispersed bimetallic surface alloys are excellent nanocomposite materials for fine-tuning the active centers of a number of homogeneous [1], heterogeneous [2–7], photo-[8, 9] and electro-catalysts [6, 10, 11]. By systematically studying appropriate model systems, it is possible to establish the connection between their structure and activity [4, 12–16]. The exceptional and enhanced catalytic properties of alloyed, bimetallic systems arises from the synergistic properties of the two metals. Indeed, the relationship between the surface free energies and work functions of the metals and their support plays a crucial role in surface and subsurface processes [17]. Furthermore, the activity and selectivity of these catalysts is influenced by the composition and morphology of the nanoparticles (NPs) [18, 19].

Pd–Au alloyed catalysts have proved to be excellent catalysts for a number of chemical processes such as the acetoxylation of ethylene to vinyl acetate [12, 20], solvent-free oxidation of primary alcohols to aldehydes [21] and direct synthesis of H_2_O_2_ from H_2_ and O_2_ [22]. Recently, it has been demonstrated that Au-core Pd-shell NPs in microbial fuel cells exhibit enhanced catalytic activity in wastewater treatment technology [23].

The formation of bimetallic NPs on reducible oxide surfaces is a rather complex process in that reduced species (such as O vacancies) on the oxide support play an influential role in the process of nanoparticle nucleation [24, 25]. The structure of the supported composite nanoparticles are largely determined by the nanoscale thermodynamics of their components and interfaces, which may lead to a large variation in their composition [26–34]. This tunability of composition offers considerable potential for enhanced selectivity and activity in catalytic applications [35, 36]. Encapsulation of supported metal NPs as a result of strong metal supported interaction (SMSI) can be expected on easily reducible oxide supports such as TiO_2_, TaO_5_, CeO_2_ and NbO for VIII.B metals (Ir, Rh, Ni, Pd and Pt) whose work function is above 5.3 eV and with a surface energy over 2 Jm^− 2^ [17].

In general, the encapsulation of the supported metal nanoparticles degrades their catalytic activity by decreasing the number of the catalytically active sites [37, 38], although SMSI enhancement of activity has also been reported [39]. Although it is possible to decorate a Au(111) surface with TiO_x_ by oxidising a Ti adlayer [40], it does not appear possible to encapsulate Au nanoparticles on a reducible support. Moreover, a Au overlayer on a VIII. B metal can strongly hinder the SMSI process [31, 32], while alloy formation at higher temperatures can also occur [41, 42].

Associated with interest in graphene like 2D materials [43], there have been a number of studies of 2D oxide nanomaterials [31, 44, 45]. The commonly accepted stacking sequence of TiO_x_ ultrathin oxide layers on transition metals is the following: M–Ti–O_x_, where M corresponds to the transition metal bonded to the oxide substrate [46, 47]. The lattice mismatch and the rotation between the transition metal’s lattice and Ti lattice creates a moiré pattern with unit cell dimensions around 1.5 nm in the case of the pinwheel structure. This periodicity coincides with the superlattice’s unit cell’s corners, creating periodically lower-surface-potential areas (break in the homogeneous surface potential) in the oxide layer, which can act as a trapping site for adsorbed metal atoms. In connection with this property, template assisted adsorption and growth of Au nanoparticles has been investigated previously in the cases of Rh(111)/TiO_x_/Au [47] and Pt(111)/TiO_x_/Au [44]. In contrast, the adsorption properties of Au on a more complex system such as encapsulated bimetallic nanoparticles have not yet been reported.

Here we report the study of the influence of the Au:Pd ratio on the core–shell composition of nanoparticles on TiO_2_(110). Furthermore, we study the morphology of submonolayer Au deposited on the TiO_x_ ultrathin oxide films formed on the supported Au–Pd nanoparticles.

## Experimental

The experiments were carried out in London using an *Omicron GmbH* variable temperature scanning tunneling microscope (STM) housed in an ultrahigh vacuum chamber (base pressure = 5 × 10^− 11^ mbar). The system was also equipped with low energy electron diffraction (LEED) optics and a retarding field Auger-electron spectrometer (AES). TiO_2_(110) single crystals (*Pi-Kem*) were prepared by cycles of argon ion sputtering (1 kV) and annealing in vacuum at 1000 K. The sample cleanliness and long range order were confirmed using AES and LEED, respectively. Pd and Au were deposited onto the as-prepared TiO_2_(110) surface at room temperature. Dosing rates were calibrated with AES. The Pd (Au) evaporation source consisted of a Pd (Au) wire wrapped on tungsten filament that was resistively heated. For the formation of the bimetallic nanoparticles, the same deposition conditions were maintained such that the coverage of Au and Pd on TiO_2_(110) could be estimated from the volume of the nanoparticles measured with STM. The coverage of Pd and Au is expressed in equivalent monolayers (MLE), defined as the surface concentration of Pd(111) and Au(111), respectively (for Pd 1 MLE ~ 1.53 × 10^15^ atoms/cm^2^, while for Au 1 MLE ~ 1.39 × 10^15^ atoms/cm^2^). STM images were recorded in constant current mode using W tips prepared by electrochemical etching and conditioned by outgassing at 500 K as well as voltage pulses in STM. The STM images were analyzed using WSxM software [48]. For determining the average height, at least one hundred nanoparticles were measured.

## Results and Discussion

### Formation of a Pd Film (8 MLE) at RT on TiO_2_(110)-(1 × 1) and the effects of the annealing up to 1000 K

**Fig. 1:**
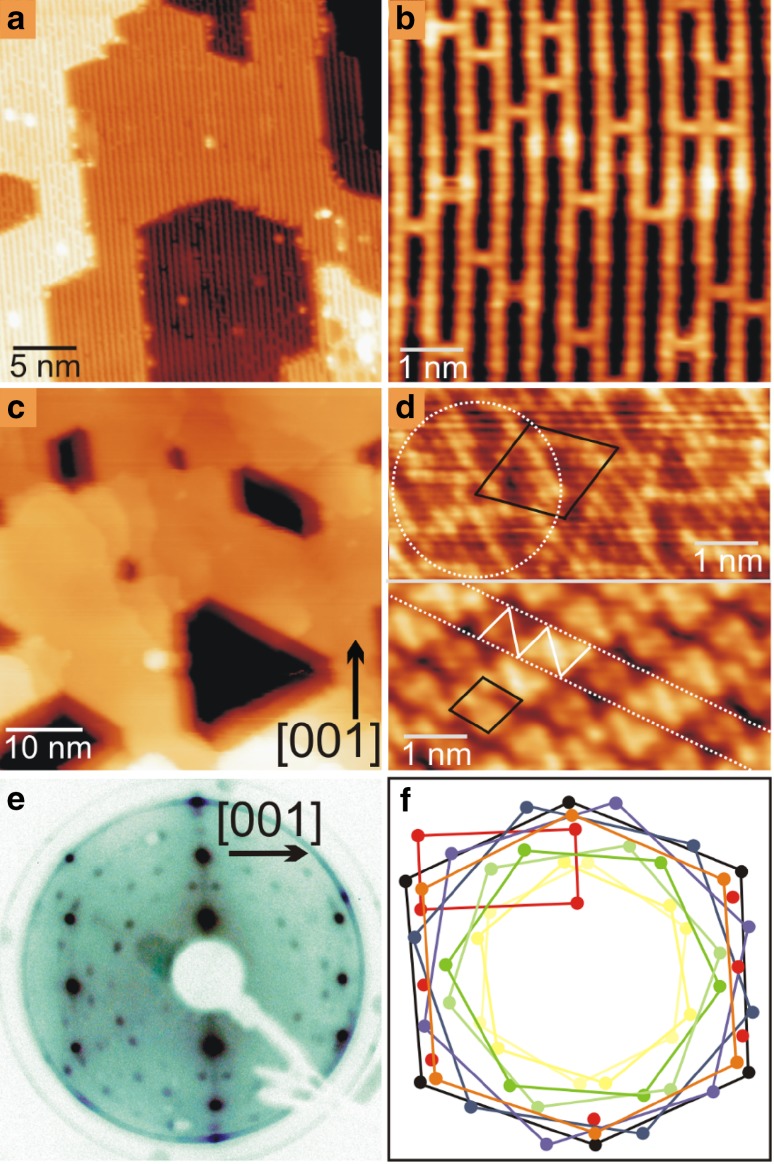
**a, b** STM images of as-prepared TiO_2_(110)-(1 × 1). **c** As **a**, after adsorption of 8 MLE Pd, followed by annealing at 973 K for 1 h. **d** Atomically resolved images from the Pd nanoparticle top facets that exhibit pinwheel (upper) and zig–zag (lower) encapsulation structures. Image sizes: **a** 30 × 30, **b** 6 × 6, **c, d** 6 × 3 nm^2^. **e** LEED pattern (*E* = 48 eV) taken from the surface in **c. f** Assignments of the LEED pattern. The red rectangle marks the unit cell of TiO_2_(110)-(1 × 1), while the black hexagon marks the unit cell of Pd(111), revealing a calculated lattice constant of 2.7 ± 0.15 Å. The orange hexagon marks the pinwheel structure, while the yellow, green and blue hexagons mark pinwheel-like structures. Scan parameters: **a**–**c** V_S_, I_T_ = + 1.5 V, 1.0 nA and **d** + 0.2 V, 5 nA

Figure 1a shows a typical STM image of the as-prepared TiO_2_(110)-(1 × 1) surface. The surface comprises large (1 × 1) terraces separated by step edges that run along the [001] and [1–11] crystallographic directions [49]. On the terrace (Fig. 1b), alternating bright and dark rows, corresponding to the rows of fivefold coordinated Ti (Ti_5c_) and twofold coordinated bridging O (O_b_) ions respectively, run along the [001] direction. Bright features that link the neighboring Ti_5c_ rows are the missing O_b_ ions, namely O_b_ vacancies (or O_b_-vacs) [50, 51].

Deposition of eight monolayer equivalents (MLE) Pd onto TiO_2_(110) results in the formation of a continuous multilayer film of Pd that dewets partially after annealing at 953 K for 1 h (Fig. 1c). The morphology of the film largely follows that of the substrate, with some quasi-hexagonal holes that develop inside the film. Atomically resolved STM images recorded from the top part of the Pd film (Fig. 1d) reveal that it is encapsulated by the well-known pinwheel and zigzag TiO_x_ structures as a result of strong metal–support interaction (SMSI) [49, 52, 53]. The pinwheel structure has a hexagonal unit cell with cell length of 1.7 ± 0.1 nm (black lines in the top part of Fig. 1d), oriented 30° with respect to the [001] direction of the TiO_2_(110) substrate, while a dotted white circle picks out the pinwheel structure. The measured interatomic distance in the pinwheel structure is 3.3 ± 0.1 Å. The zig–zag TiO_x_ structure has a rhombic unit cell with cell parameters of 0.8 × 0.66 nm^2^ (black lines, bottom part of Fig. 1d). The interatomic distances in the zigzag structure are 2.9 ± 0.1 and 3.3 ± 0.1 Å. The trough and the glide plane of the zigzag is marked with white dotted lines, while the continuous white lines depict the zigzag structure in the bottom part of Fig. 1d. These results are in a good agreement with earlier work [54, 55].

We also performed LEED on the same surface. As shown in Fig. 1e, f, the LEED pattern is composed of diffraction spots of the TiO_2_(110) substrate (red squares in Fig. 1f) and those of the (111) top-facet of the Pd multilayers (black hexagons), from which the nearest neighbour distance of Pd atoms of the Pd(111) top-facet was calculated to be 2.70 ± 0.15 Å. In addition, the LEED pattern also has diffraction spots that originate from different TiO_x_ encapsulation layers. These include the diffraction spots originating from the pinwheel structure (orange hexagons, rotated by ± 3° with respective to the Pd(111) pattern), as well as the diffraction patterns that we attribute to other pinwheel-like structures (blue, green, and yellow respectively in Fig. 1f). The unit-cell parameters of the pinwheel as well as of pinwheel-like structures are listed in Table 1. Although we observed the zigzag TiO_x_ structure in STM, its corresponding diffraction spots were absent from the LEED pattern. This is probably due to the presence of glide-planes, as previously described in the work of Bennett et al. [54].

**Table 1:** Interatomic distances calculated from the LEED pattern in Fig. 1

Structure notation	Nearest neighbor distance (nm)	Rotation angles relative to Pd(111) (˚)
Pd(111)-(Black spots)	0.27 ± 0.015	–
Pinwheel-(Orange)	0.33 ± 0.010	± 3
Pinwheel-like-(Blue)	0.31 ± 0.010	± 15
Pinwheel-like-(Green)	0.35 ± 0.020	± 30
Pinwheel-like-(Yellow)	0.48 ± 0.020	± 3

### Pd (~ 5 MLE) and Au (~ 1 MLE) double layer deposited on TiO_2_(110)-(1 × 1) at room temperature and the effects of annealing

**Fig. 2:**
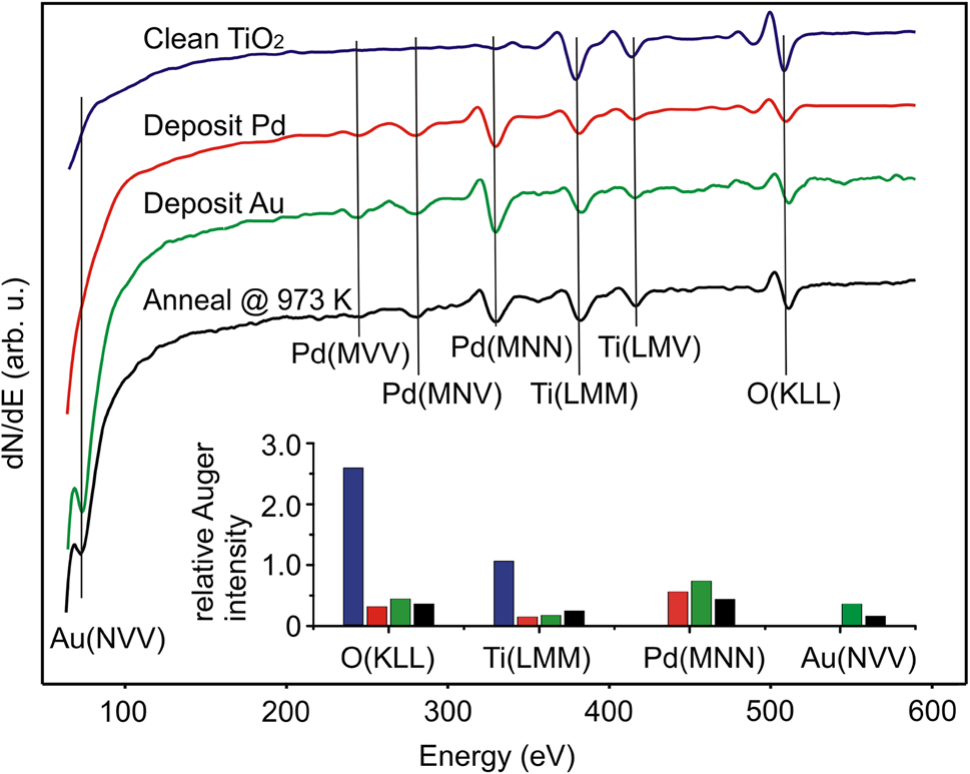
Auger electron spectra taken from four different surfaces: (Blue) clean TiO_2_(110), (Red) TiO_2_ after adsorption of 5 MLE Pd at 300 K; (Green), TiO_2_ after adsorption of 5 MLE Pd followed by 1 MLE Au at 300 K, and (black) the (5 MLE Pd + 1 MLE Au)/TiO_2_ surface after annealing at 973 K for 20 min. Inset: relative intensities of different Auger transition signals for the four different surfaces

As shown in Fig. 1 and previous work [24, 54, 55], Pd nanoparticles supported on TiO_2_(110) become encapsulated by an ultrathin TiO_x_ layer when the Pd/TiO_2_ surface is annealed at > 873 K. Here we investigate whether incorporating Au into the Pd nanoparticles affects the encapsulation process. This might be expected because Au is more inert as a metal, and has a lower work function (~ 5.3 eV) and surface free energy (~ 1.51 Jm^− 2^) compared to Pd (5.6 eV and 2.01 Jm^− 2^). The experiment was performed by sequentially depositing 5 MLE of Pd and then 1 MLE of Au on TiO_2_(110) at room temperature, followed by annealing at 973 K for 20 min. AES spectra and STM images were recorded after each of the surface treatment steps. Figure 2 shows a number of AES spectra taken after each of the aforementioned surface treatment was performed, with the inset of which showing the relative intensities of the Auger transition signals of four different elements (O, Ti, Pd and Au), all normalized with respect to the intensity of the Ti(*LMM*) signal recorded from the clean TiO_2_(110) surface. As shown in Fig. 2, the spectrum taken from the clean TiO_2_(110) surface is characterized by three peaks, one of which is at 509 eV, the other two being at 384 and 416 eV. The peak at 509 eV corresponds to the *KLL* Auger transition of O, while the other two peaks at 384 and 416 eV correspond to the *LMM* and *LMV* Ti Auger transitions, respectively. Then 5 ML of Pd and 1 ML of Au were sequentially deposited onto the TiO_2_(110) substrate at room temperature (RT).

Following the deposition of Pd (red), the intensities of the Auger transitions of Ti and O are reduced by a factor of around five, while three new peaks at 243, 279 and 330 eV appear. They are attributed to the *MVV, MNV*, and *MNN* Auger transitions of Pd. Deposition of Au (green) onto this surface leads to no detectable change in the peak intensities from Ti, O and Pd. However, a new peak appears at 73 eV kinetic energy, which we attribute to the *NVV* Auger transition of Au. The surface was then annealed at 973 K for 20 min. As shown in the resulting spectrum in Fig. 2 (black), this results in a noticeable recovery of the Auger signals from the TiO_2_(110) substrate, which we attribute to sintering of the Pd–Au layers. There is also a sharp decrease of the Au(*NVV*) signal (by 60%). In contrast, the Pd MNN only decreases by 40%. There are several thermally induced phenomena that can result in a greater decrease in the Au(NVV) signal: evaporation of Au from the surface, Au–Pd alloy formation, and diffusion of Au into the Pd multilayers. It has been reported that at < 1000 K Au does not evaporate from rutile TiO_2_ [31, 33] or alloy with Pd [2]. On this basis we attribute the decrease in the Auger signal of Au to the diffusion of Au into the Pd, which in turn leads to the formation of Au–Pd alloy core, Pd-shell supported nanoparticles. Although this interpretation is rather surprising when taking into account the fact that Au has a lower surface free energy (1.51 Jm^− 2^) compared to Pd (2.01 Jm^− 2^) [56], it has also been evidenced in a recent ion scattering spectroscopy (ISS) and X-ray photoelectron spectroscopy (XPS) study [42]. Moreover, in earlier work Au was found to diffuse into a Pd(111) single crystal [2]. In the ISS/XPS work, 5 ML Pd and 0.4 ML Au were sequentially deposited onto TiO_2_(110) at room temperature before annealing in the range 473–873 K. Although the Au ISS signal disappears at 773 K, XPS shows that the total amount of Au is maintained. This therefore suggests the formation of Au–Pd core Pd shell NPs. Further annealing to above 773 K leads to encapsulation of these bimetallic NPs with an atomically thick TiO_x_ layer as a result of SMSI [42].

**Fig. 3:**
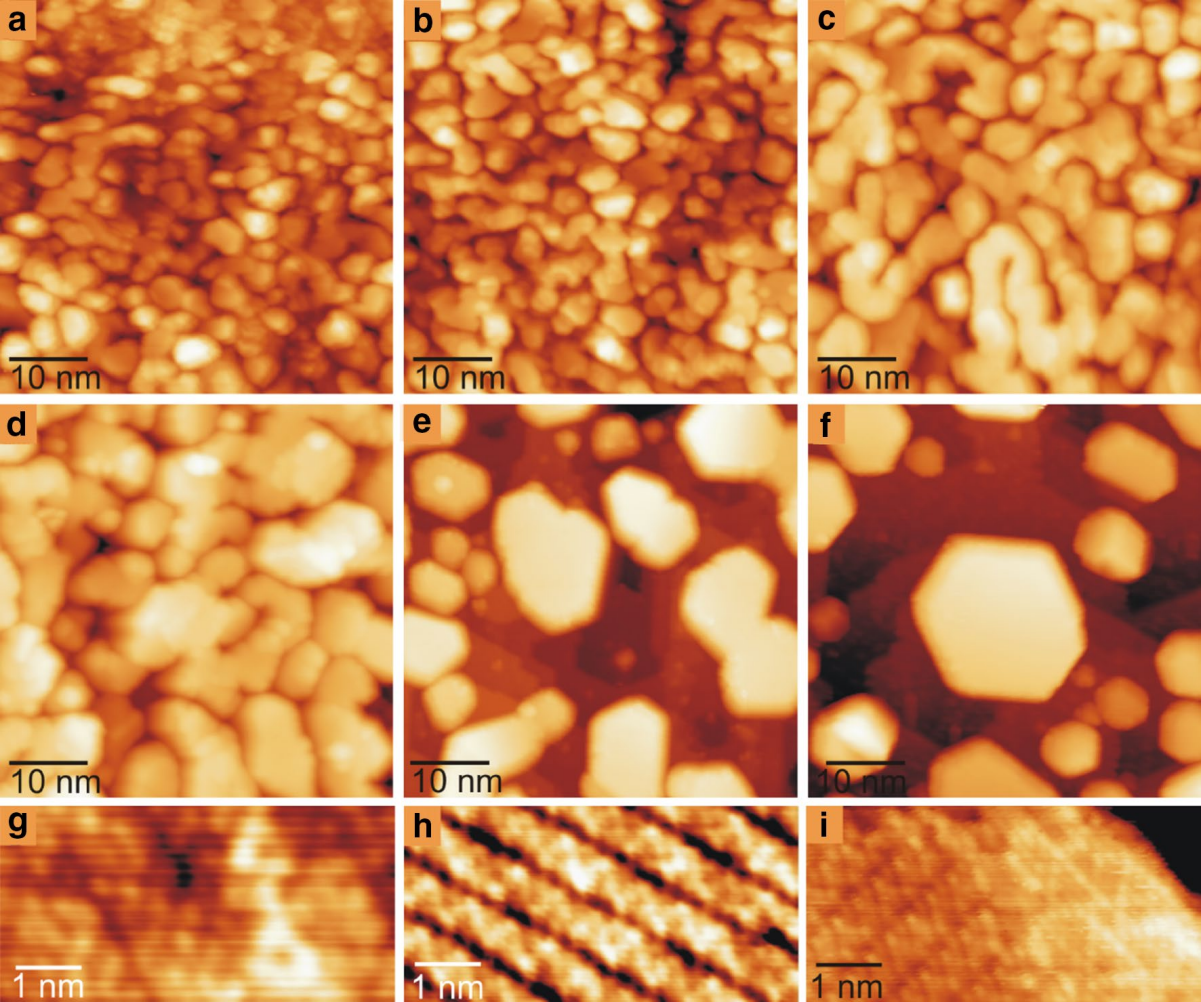
50 × 50 nm^2^ STM images recorded from TiO_2_(110) after sequential deposition of **a** 5 MLE Pd and **b** 1MLE Au at 298 K, and **c–f** annealing at **c** 573, **d** 773, **e** 873 and **f** 973 K, for 20 min. at each temperature. **g**–**i** 6 × 3 nm^2^ atomically resolved STM images of the top-facet of one of the Au–Pd nanoparticles shown in **d**–**f**, respectively. All STM images were recorded at room temperature. Scan parameters: (**a**–**f**) V_S_, I_T_ = + 1.2 V, 1.0 nA and **g, h** + 0.6 V, 3.0 nA

Here STM was also employed in order to obtain a clearer picture about the thermally induced material transport processes on this bimetallic system. As above, 5 ML Pd was first loaded onto the clean TiO_2_(110) substrate at RT. As shown in Fig. 3a, this resulted in a continuous Pd multilayer forming over the entire TiO_2_(110) substrate. A closer look at the Pd multilayer reveals that it is composed of a dense population of Pd grains, which were measured to have an averaged diameter of ~ 5 nm and height of ~ 1.2 nm. Subsequent deposition of 1 ML Au onto the Pd/TiO_2_(110) surface did not result in any noticeable change in the surface morphology as observed by STM (Fig. 3b). This is to be expected since the amount of Au added to the system only accounts for 16% of the total number of metal atoms deposited. Annealing the surface at 573 K for 20 min results in sintering (Fig. 3c). Increasing the anneal temperature to ~ 773 K leads to further dewetting of the Au–Pd multilayer, which exposes small areas of the TiO_2_ substrate (Fig. 3d). Annealing the surface to higher temperatures between 873 and 973 K facilities coalescence and/or Ostwald ripening, leading to the formation of separate, pseudo-hexagonal Au–Pd bimetallic nanoparticles supported on the TiO_2_(110) substrate (Fig. 3e, f). These nanoparticles have an averaged diameter of ~ 15 nm and height of ~ 2.5 nm.

Corresponding high resolution STM images of the nanoparticles (Fig. 3h, i), reveal evidence of encapsulation with a zigzag-like TiO_x_ layer. Indeed Fig. 3g, corresponding to an anneal temperature of ~ 773 K, reveals some traces of the development of the zigzag and pinwheel TiO_x_ structures. This, along with the absence of any Au related features or discontinuities in the encapsulating oxide frameworks, supports the formation of Au/Pd-core, Pd-shell nanoparticles at temperatures of 773–973 K. The formation of this type of structure must arise from kinetic factors, as suggested previously [42, 57–59]. On the other hand, there also has to be an equilibrium concentration between the two metals within the Au–Pd mixture above which Pd no longer dissolves Au in its bulk. On this basis, we believe that Au/Pd core, Au-shell bimetallic NPs can be formed when the relative Pd/Au concentration is above such a threshold.

### Pd (~ 3 MLE) and Au (~ 2 MLE) double layer deposited on TiO_2_(110)-(1 × 1) at room temperature and the effects of annealing

**Fig. 4:**
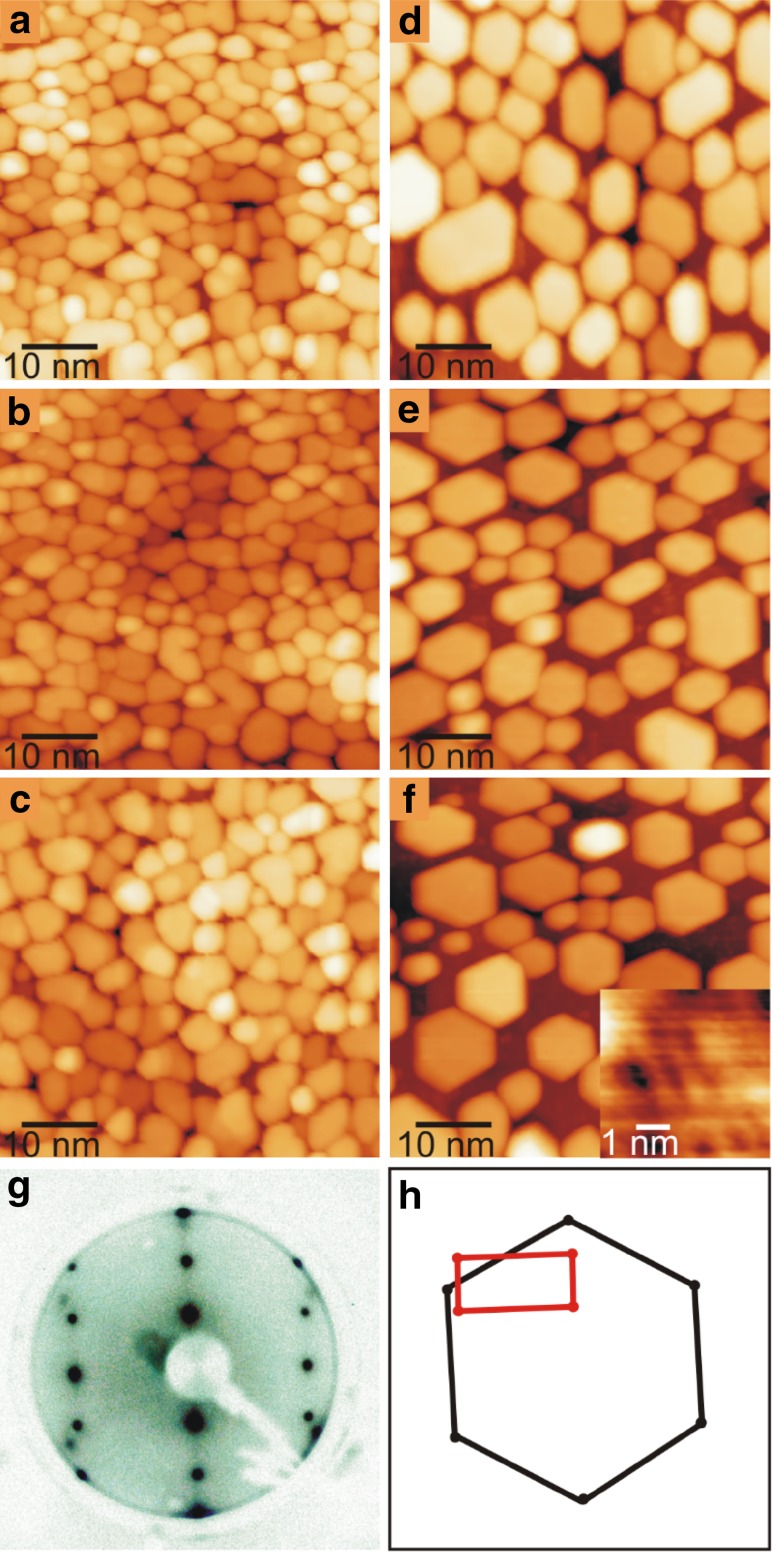
**a** STM image of TiO_2_(110)-(1 × 1) after adsorption of 3 MLE Pd at 300 K. **b** As **a**, after additional adsorption of 2 MLE Au at 300 K. **c**–**f** STM images after the surface in **b** was sequentially annealed to **c** 573 K, **d** 773 K, **e** 873 K and **f** 973 K for 20 min. at each temperature. Inset in **f** high-resolution image recorded from the top facet of one of the Pd-core, Au-shell nanoparticles. Image sizes: **a**–**f** 50 × 50 nm^2^, inset of **f** 6 × 6 nm^2^. **g** LEED pattern taken from the surface in **f** at an electron energy of 87 eV. **h** Assignment of LEED pattern in **g**. Red squares mark the TiO_2_(110)-(1 × 1) unit cell, black hexagons mark the Au(111) unit cell. This gives a calculated nearest neighbour distance of 2.9 ± 0.1 Å. Scan parameters: **a**–f V_S_, I_T_ = + 1.5 V, 0.1 nA and inset of **f** + 0.2 V, 5.0 nA

To test this idea, we sequentially deposited a reduced amount of Pd (3 MLE) and a doubled amount of Au (2 MLE) onto TiO_2_(110) at room temperature and investigated the effect of annealing as before. As shown in Fig. 4a, following the deposition of 3 MLE Pd at room temperature a large number of small Pd nanoparticles are formed across the TiO_2_(110) substrate. These Pd nanoparticles have a mean inter-particle distance of 0.8 ± 0.3 nm and average height of 0.9 ± 0.1 nm. Adsorption of 2 MLE of Au onto this surface leads to an increase in the average particle height to 1.2 ± 0.2 nm, while the mean inter-particle distance remains unchanged (Fig. 4b). The average height was measured from around 100 nanoparticles. This indicates that the Au atoms nucleate on the Pd nanoparticles, which is consistent with the behavior expected for a Pd(111) top facet of the nanoparticle [60].

STM images (Fig. 4c–f) show that the morphology changes on annealing sequentially from 573 to 973 K are more gradual compared with the 5 MLE Pd: 1 MLE Au mixture described above. As the anneal temperature increases, small Au–Pd bimetallic nanoparticles ripen/coalesce, resulting in the formation of larger, more well-defined pseudo-hexagonal nanoparticles on the TiO_2_ support. The LEED pattern (Fig. 4g, h) from the surface annealed to 973 K is consistent with a (111) termination of the bimetallic nanoparticles. The corresponding nearest neighbor distance on the (111) top-facet is 2.9 ± 0.1 Å. This value is much larger than the lattice parameter of Pd(111) (2.75 Å), but very close to that of Au(111) (2.89 Å). This points to a Au(111) top layer of the nanoparticles.

The LEED pattern of the 973 K annealed surface shows no trace of the signature pattern of the pinwheel and zigzag structures, suggesting the absence of encapsulation. STM is consistent with this picture. As shown in the inset of Fig. 4f, the high resolution STM image taken from the top-facet of one of the bimetallic NPs exhibits an STM contrast that is much weaker in corrugation and very different from those of the pinwheel- and zigzag types of TiO_x_ layer (Figs. 1d, 3g–i). We speculate that the image contrast arises from sub-surface alloy ordering. The absence of encapsulation, and Au(111) termination of the nanoparticles at this higher Au concentration is in line with our expectations. It is also consistent with earlier work where it was reported that a thick layer of Au covering Rh nanoparticles on TiO_2_(110) hinders the diffusion of Ti and O species onto the top facet up to ~ 900 K [32].

**Fig. 5:**
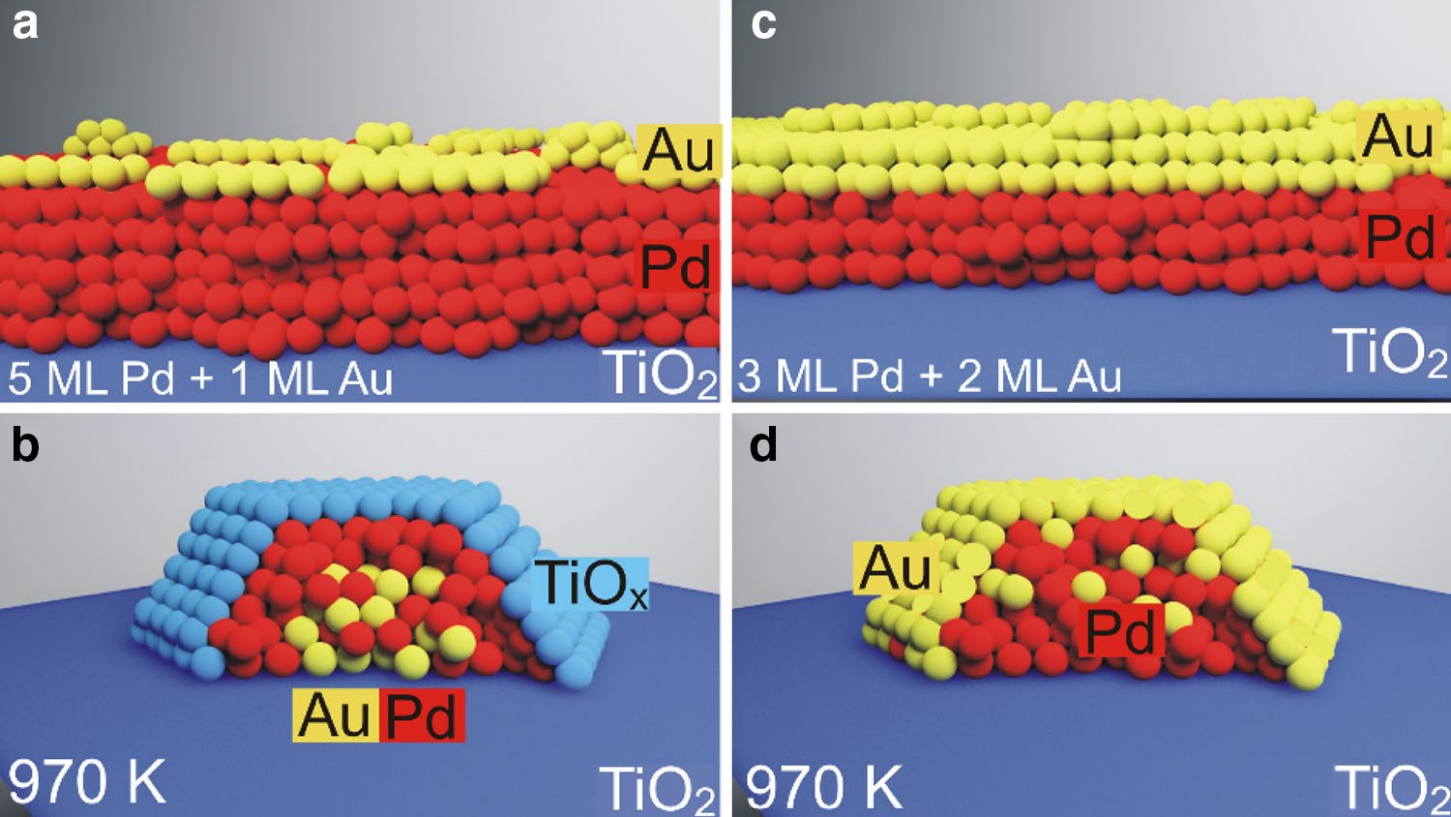
Schematic ball models of the alloying and encapsulation processes of Au/Pd overlayers: **a** TiO_2_(110) afer adsorption of 5 MLE Pd followed by 1 MLE Au at 300 K. **b** As **a**, after sintering caused by annealing at 973 K for 20 min. **c** TiO_2_(110) after adsorption of 3 MLE Pd followed by 2 MLE Au at 300 K. **d** As **c**, after sintering caused by annealing at 973 K for 20 min

A summary of our conclusions regarding the effect of annealing Au/Pd nanoparticles is shown in Fig. 5. In the low Au-content regime (Fig. 5a. b), the nanoparticle has a bimetallic core and Pd shell, and as a result is encapsulated by the ordered TiO_x_ structure after annealing. However, in the regime where the Au content is high (Fig. 5c, d), the nanoparticle core cannot dissolve more Au, and as a result the remaining Au atoms stay in the nanoparticle shell. The top facet appears to be essentially Au(111), which lowers the surface free energy and hinders SMSI of the nanoparticles.

### Template assisted adsorption, growth and thermal stability of Au nanoparticles on TiO_x_

**Fig. 6:**
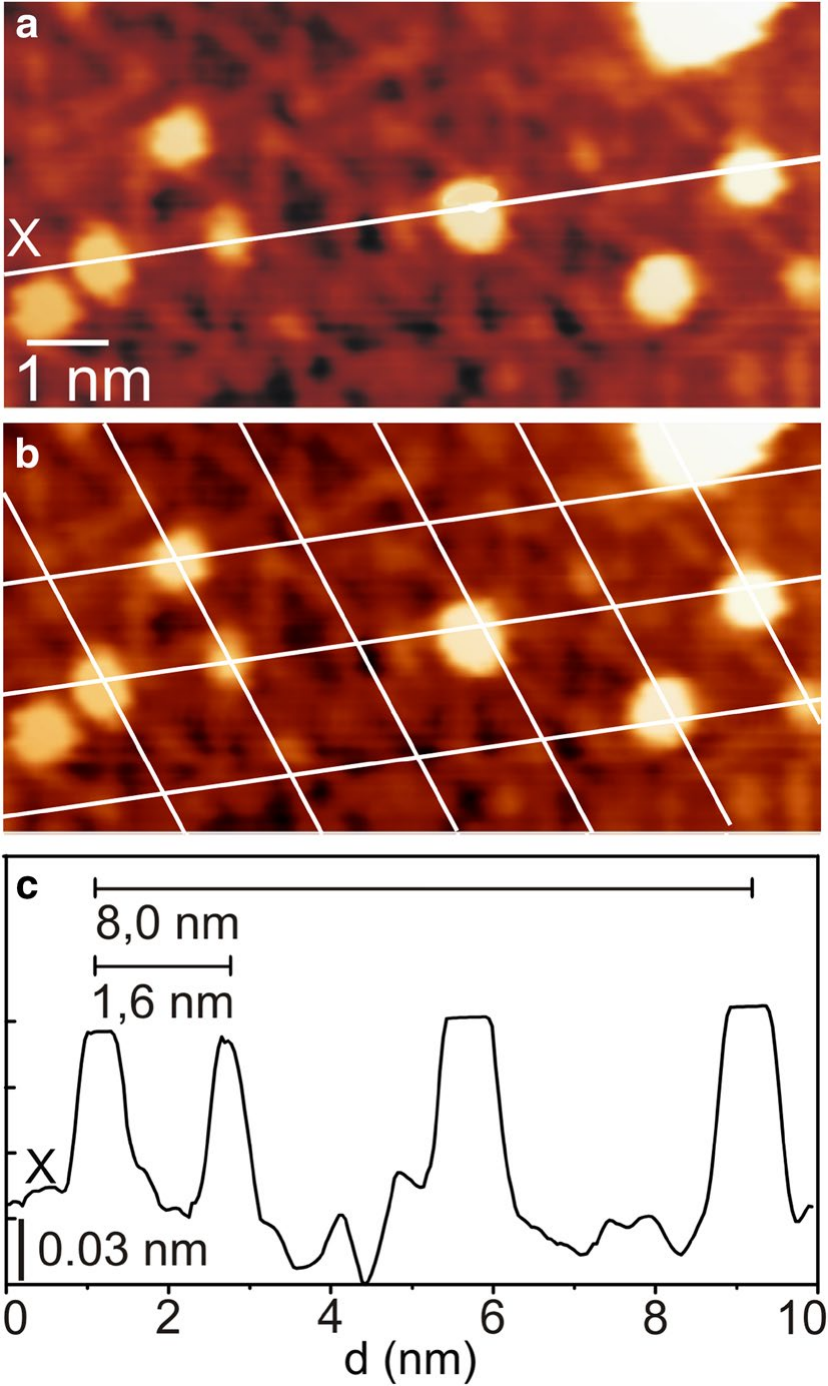
**a** 10 × 5 nm^2^ STM image from the pinwheel encapsulating TiO_x_ layer after adsorption of 0.1 MLE Au at 300 K (V_S_, I_T_ = + 0.6 V, 1.0 nA). The encapsulation layer was formed on TiO_2_(110) after adsorption of 5 MLE Pd and 1 MLE Au followed by annealing to 973 K for 20 min. **b** As **a**, with white lines marking the unit cells of the pinwheel structure. These images reveal the pico-holes in the pinwheel structure to be the preferential adsorption sites for the Au clusters comprising 3–10 Au atoms. **c** Line plot taken along the line marked in **a**, showing a nearest neighbour distance of 1.6 ± 0.1 nm between the Au clusters

One of the most intriguing aspects of ultrathin oxide layers is the possibility to use them as templates for metal adsorption to form uniform, periodically located metal nanoparticles on the oxide layer. This sort of behavior arises from the presence of more energetically favourable adsorption sites at the inter-corner regions (so-called pico-holes) of the pinwheel TiO_x_ structure, which are not necessarily atomic vacancies in that oxide layer [45–47, 61]. Figure 6a shows an STM image of the pinwheel oxide layer following exposure of 0.1 MLE Au at RT. As indicated by the line plot in Fig. 6c, the Au species on the pinwheel structure have a measured diameter of 23 ± 5 Å and height of 1.1 ± 0.2 Å. In addition, by overlaying the image with a grid marking the lattice of the pinwheel structure (Fig. 6b), we can easily see that ~ 90% of the Au species adsorb at the pico-holes of the pinwheel structure, revealing a nearest neighbour distance of 1.6 ± 0.1 nm between the Au species. This suggests that the pico-holes of the pinwheel type oxide layer act as trapping sites for the impinging Au atoms, with bonding to the underlying metal at room temperature. Our findings hence agree extremely well with those reported by the Berkó group, who observed the Au species on the pinwheel type TiO_x_ layer on the Rh(111) facet to exhibit very similar behavior at slightly higher deposition temperature (400–500 K) [31, 47]. Also, it is noteworthy that unlike the Au species on the pinwheel type TiO_x_ layer, those nucleating on the zigzag type TiO_x_ layer do not show any preference regarding the adsorption site.

**Fig. 7:**
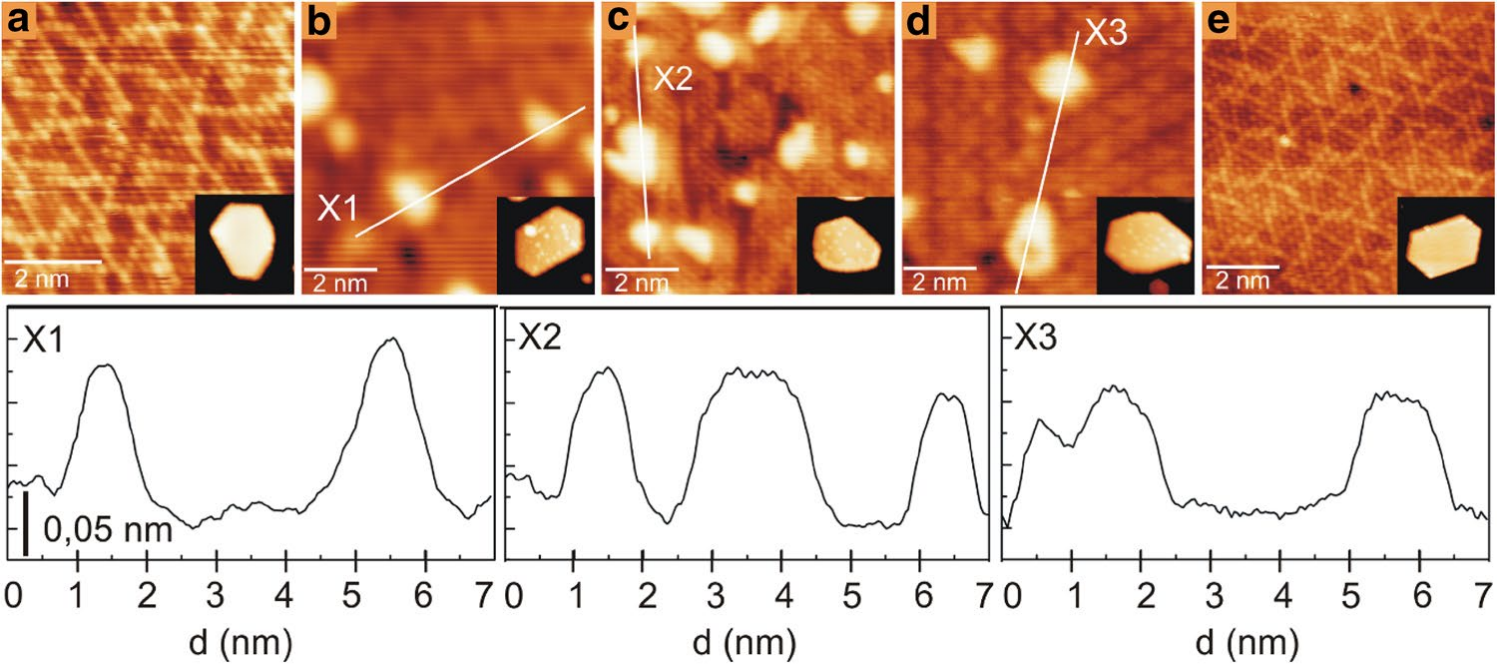
The effect of annealing on the size and distribution of Au clusters deposited onto the TiO_x_ encapsulation layer. **a, b** STM images recorded **a** before and **b** after deposition of 0.1 MLE Au on the encapsulating TiO_x_ layer of the supported Au–Pd bimetallic nanoparticles shown in Fig. 6 at 300 K. **c**–**e** As **d**, after the surface was sequentially annealed at **c** 573, **d** 873 and **e** 973 K for 20 min. at each temperature. Image size: **a, b** 6 × 6 and **c**–**e** 8 × 8 nm^2^. Insets of **a**–**e**: the supported Au–Pd bimetallic nanoparticles on which the STM images in **a**–**e** were recorded. Average width and height of the NPs is 25 nm and 2.0 nm. Scan parameters: **a**–**e** V_S_, I_T_ = + 0.1 V, 3.0 nA and insets of **a–e** + 1.3 V, 0.1 nA

We annealed the Au nanoparticles on TiO_x_ to explore their thermal stability. Figure 7a–e show high resolution STM images of the encapsulating TiO_x_ layer formed on the Au–Pd bimetallic nanoparticles taken before (Fig. 7a) and after deposition of 0.1 MLE of Au at room temperature (Fig. 7b), and those taken after annealing at different temperatures (Fig. 7c–e). The insets in Fig. 7c–e show the morphology of the nanoparticles. As shown very clearly by the line-plots (i.e. line-plots X1 to X3 in Fig. 7f), the Au species retain more or less their original size and shape even after surface annealing at ~ 873 K, evidencing their apparent thermal stability. However, there is an apparent reduction in the number density of Au nanoparticles with increasing temperature, reaching 20% of initial density at 873 K before the nanoparticles completely disappear at 973 K. In contrast, Au nanoparticles directly bound to the TiO_2_ substrate are still present after annealing at 973 K (image not shown), which we attribute to their high desorption temperature from rutile [62].

In order to interpret our observation it is worth comparing our results with those by Berkó group [31]. In their work, 1 MLE Au was deposited at 500 K onto the TiO_x_ layer formed on the (111) top facets of the Rh nanoparticles, with subsequent annealing up to 1050 K. In this earlier work it was observed that the Au species on the oxide layer are 3D in shape at lower temperatures. However, Ostwald-ripening starts to dominate as the temperature increases. At even higher temperature the Au nanoparticles start to spread out, forming a continuous, two-dimensional (2D) layer of Au across the entire oxide layer. This Au layer then disappears from the oxide layer at above 1000 K, which the authors attributed to the desorption of Au from the surface on the basis that the dissolution of Au into Rh is negligible [41]. In the present case where Au is a miscible metal with Pd, in addition to desorption of Au from the surface, the disappearance of the Au species from the oxide layer can also originate from the dissolution of the Au species into the bulk of the Au–Pd bimetallic nanoparticles [42].

## Conclusions

Using STM, AES and LEED we have demonstrated that encapsulation of Au–Pd bimetallic nanoparticles on rutile TiO_2_ can be prevented by increasing the relative Au concentration. Such enrichment of Au leads to the formation of nanoparticles with a bimetallic core and Au-rich shell, which has a lower surface free energy and therefore is much less prone to SMSI.

We have also studied the room temperature adsorption behavior of Au on the pinwheel- and zigzag- type TiO_x_ layers formed on the Au–Pd bimetallic nanoparticles. In STM, we found that the Au species preferentially adsorb at the pico-holes of the pinwheel structure, thus confirming the template-assisted growth mode as proposed by the Berkó group, while on the zigzag structure no preferential adsorption site of Au was detected. While the morphology of these Au species are stable at increasing temperatures, they vanish from the oxide layer completely at ~ 950 K, which we attribute to the dissolution of Au into the bulk, or their migration to the subsurface region of the nanoparticles.
